# The Traveling Libby Legacy: Minnesota Community Exhibits Nonoccupational Health Impacts Consistent with Asbestos Damage

**DOI:** 10.1289/ehp.120-a35a

**Published:** 2012-01-01

**Authors:** Tanya Tillett

**Affiliations:** Tanya Tillett, MA, of Durham, NC, is a staff writer/editor for *EHP*. She has been on the *EHP* staff since 2000 and has represented the journal at national and international conferences.

Adverse health effects of asbestos have been documented worldwide for the better part of the last half-century, with a large number of studies focused on occupational exposures received during the mining and processing of vermiculite ore. A new study reports on nonoccupational health effects resulting from community exposures to Libby vermiculite ore processed at the Western Minerals/W.R. Grace (WM/WRG) facility in Minneapolis, Minnesota [*EHP* 120(1):44–49; Alexander et al.].

The WM/WRG plant is located in a residential neighborhood that includes a mix of single- and multifamily homes as well as schools and churches. During its years of operation from 1938 to 1989, residents were allowed to haul away waste rock for use in their yards, and children played on piles of waste rock outside the plant.

Previous studies indicated an association between changes in lung function––a risk factor for asbestos-related disease—in children who reported playing on piles of waste rock in Libby, Montana, where this vermiculite ore was mined. In the current study, researchers analyzed radiographs of the lungs of 461 Minneapolis community members who were at risk for exposure to the WM/WRG vermiculite prior to 1980 (this date ensured an adequate latency period for any asbestos-related effects to manifest). The participants were part of the original Northeast Minneapolis Community Vermiculite Investigation cohort initiated by the Minnesota Department of Health and the U.S. Agency for Toxic Substances and Disease Registry.

**Figure:**
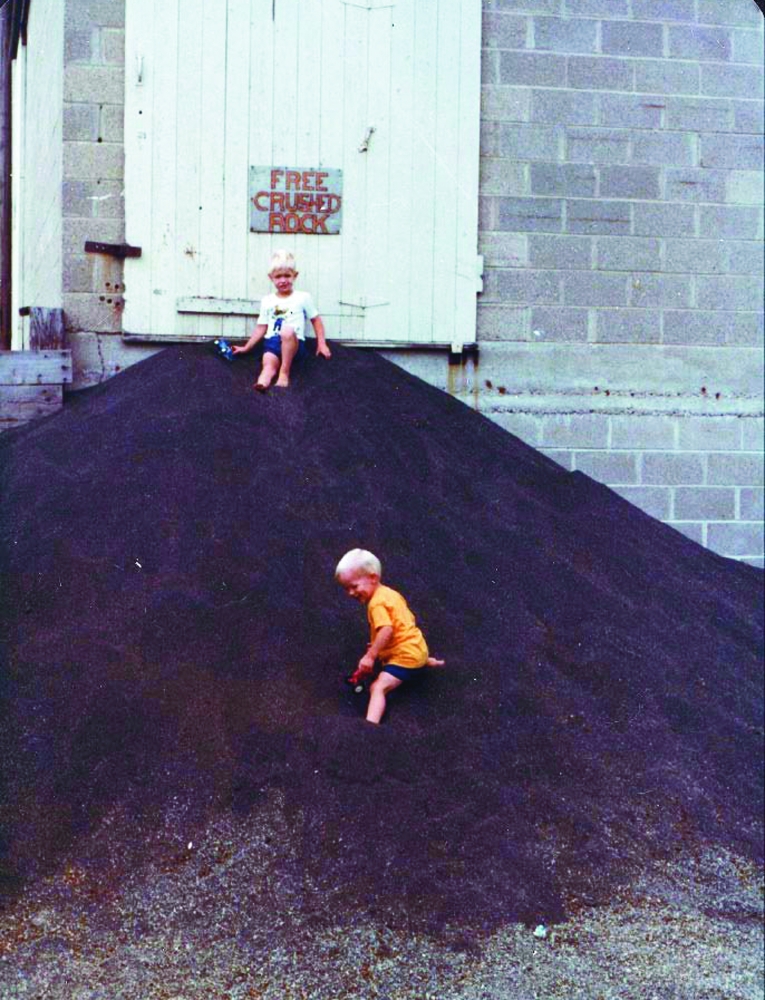
Archival photo of children playing on waste rock at the WM/WRG plant. Minnesota Department of Health

Participants’ background asbestos exposures were estimated by coupling residential history with air dispersion and deposition models of the asbestos emitted from the plant when it was in operation. The researchers also asked participants about activity-based routes of exposure, such as having played as a child on the piles of waste rock outside the plant.

Nearly half the participants reported engaging in at least one activity that brought them into contact with the waste material, and 39% reported having played on the waste rock piles. Radiographic evidence showed a 10.8% prevalence of pleural abnormalities consistent with pneumonoconiosis—evidence of asbestos-induced changes in the lungs—as determined by consensus of two radiologists certified to evaluate X rays for the effects of asbestos. Participants with pleural abnormalities were more likely to be male and were older, and those who reported frequently playing on waste rock piles had twice the prevalence of pleural abnormalities compared with those who reported never playing there. Pleural abnormalities also were more strongly associated with cumulative exposure estimated to have been acquired over many years than with higher intermittent peak exposures.

Study limitations included small population size, imprecise estimations of activities leading to exposure, and analysis of only a single chest radiograph for each study subject. However, the results suggest that health effects associated with asbestos exposure may occur at lower levels than previously believed, pointing to the need for further research on the effects of long-term, low-level, and nonoccupational asbestos exposures.

